# Preparation, Chemical Composition, and Optical Properties of (*β*–Ga_2_O_3_ Composite Thin Films)/(GaS_x_Se_1−x_ Lamellar Solid Solutions) Nanostructures

**DOI:** 10.3390/nano13142052

**Published:** 2023-07-11

**Authors:** Veaceslav Sprincean, Liviu Leontie, Iuliana Caraman, Oleg Lupan, Rainer Adeling, Silviu Gurlui, Aurelian Carlescu, Corneliu Doroftei, Mihail Caraman

**Affiliations:** 1 Faculty of Physics and Engineering, Moldova State University, 60 Alexei Mateevici Str., MD-2009 Chisinau, Moldova; 2 Faculty of Physics, Alexandru Ioan Cuza University of Iasi, Bulevardul Carol I, Nr. 11, RO-700506 Iasi, Romania; 3 Center for Nanotechnology and Nanosensors, Department of Microelectronics and Biomedical Engineering, Technical University of Moldova, 168, Stefan cel Mare Av., MD-2004 Chisinau, Moldova; 4 Functional Nanomaterials, Faculty of Engineering, Institute for Materials Science, Kiel University, Kaiserstr. 2, D-24143 Kiel, Germany; 5 Integrated Center for Studies in Environmental Science for The North-East Region (CERNESIM), Department of Exact Sciences, Institute of Interdisciplinary Research, Alexandru Ioan Cuza University of Iasi, RO-700506 Iasi, Romania

**Keywords:** chalcogenides, solid solutions, Gallium(III) trioxide, thin films, single crystals, optical properties, photoluminescence, photosensitivity

## Abstract

GaS_x_Se_1−x_ solid solutions are layered semiconductors with a band gap between 2.0 and 2.6 eV. Their single crystals are formed by planar packings of S/Se-Ga-Ga-S/Se type, with weak polarization bonds between them, which allows obtaining, by splitting, plan-parallel lamellae with atomically smooth surfaces. By heat treatment in a normal or water vapor-enriched atmosphere, their plates are covered with a layer consisting of *β*–Ga_2_O_3_ nanowires/nanoribbons. In this work, the elemental and chemical composition, surface morphology, as well as optical, photoluminescent, and photoelectric properties of *β*–Ga_2_O_3_ layer formed on GaS_x_Se_1−x_ (0 ≤ x ≤ 1) solid solutions (as substrate) are studied. The correlation is made between the composition (x) of the primary material, technological preparation conditions of the oxide-semiconducting layer, and the optical, photoelectric, and photoluminescent properties of *β*–Ga_2_O_3_ (nanosized layers)/GaS_x_Se_1−x_ structures. From the analysis of the fundamental absorption edge, photoluminescence, and photoconductivity, the character of the optical transitions and the optical band gap in the range of 4.5–4.8 eV were determined, as well as the mechanisms behind blue-green photoluminescence and photoconductivity in the fundamental absorption band region. The photoluminescence bands in the blue-green region are characteristic of *β*–Ga_2_O_3_ nanowires/nanolamellae structures. The photoconductivity of *β*–Ga_2_O_3_ structures on GaS_x_Se_1−x_ solid solution substrate is determined by their strong fundamental absorption. As synthesized structures hold promise for potential applications in UV receivers, UV-C sources, gas sensors, as well as photocatalytic decomposition of water and organic pollutants.

## 1. Introduction

Gallium oxide (Ga_2_O_3_) is an ultra-wide band gap emerging semiconductor material, showing a well-marked polymorphism [1,2]. Currently, there are six confirmed Ga_2_O_3_ polymorphs with different crystal structures and crystallization temperatures: *α*–Ga_2_O_3_ with rhomboidal lattice, *β*–Ga_2_O_3_—monoclinic, *γ*–Ga_2_O_3_—cubic defective spinel-type structure, *δ*–Ga_2_O_3_—cubic, *ε*–Ga_2_O_3_—orthorhombic, and *k*–Ga_2_O_3_ polytype, also with orthorhombic lattice [3,4,5,6]. In particular, *k*–polytype was identified in *β*–Ga_2_O_3_ layers subjected to energetic ion bombardment [6,7,8]. 

At temperatures greater than 870 °C, the *α*, *γ*, *δ*, and *ε* phases change to monoclinic *β*–Ga_2_O_3_, with stable structure and physical properties through the whole temperature range up to the melting point [7,8,9].

The *β*–Ga_2_O_3_ is an *n*-type semiconductor with an ultra-wide energy band gap (4.9 eV), displaying considerable application prospects in ultraviolet (UV) optoelectronics [10,11,12,13] and high-performance electronic devices [14,15,16]. Recent studies have demonstrated that micro- and nanostructured *β*–Ga_2_O_3_ and related nanocomposites are promising materials for gas sensing applications and photocatalytic degradation of hazardous organic pollutants. In [17], the *β*–Ga_2_O_3_/Al_2_O_3_ nanocomposite was synthesized by a hydrothermal method with further calcination of Al(NO_3_)_3_·9H_2_O and Ga(NO_3_)_3_·xH_2_O compositions. The tested response of this composite material on exposure to NO_x_ (about 100 ppm concentration) was ~58%. Nanocomposites with nanostructured oxide semiconductors *β*–Ga_2_O_3_, SnO, or *β*–Ga_2_O_3_/reduced graphene oxide (rGO) exhibit high sensitivity to molecular gases (O_2_, H_2_), along with flammable and toxic chemical compounds, such as H_2_S, CO_2_, NH_3_, etc. [18,19].

The critical breakdown electric field of semiconductor material is an important physical parameter, determining the technical and application characteristics of electronic devices (diodes, transistors, switches, etc.). For gallium oxide, a critical field with the value f 8 MV/cm was reported in [20]. By doping *β*–Ga_2_O_3_ with Zn, it can be increased up to 13.2 MV/cm. High values of the breakdown field determine the technical power parameters of field effect transistors [21,22].

The application area of wide-gap semiconductors is enlarged with the transition from bulk single crystals to micro- and nanocrystals. Several manufacturing technologies of *β*–Ga_2_O_3_ nanoformations (nanowires, nanoribbons, nanoparticles) are known. An extensive review of these methods is presented in the recent paper [23]. Nanostructured *β*–Ga_2_O_3_ was obtained in [24,25] by heat treatment of GaN powder and plates in nitrogen (N) flow at temperatures in the range of 850–1100 °C. In [24], obtaining, through the same technological procedures, micrometer-sized GaN grains coated with *β*–Ga_2_O_3_ micro- and nanoformations was reported.

Group-III monochalcogenides, MX (M = Ga, In, X = S, Se, Te), belonging to the class of lamellar III-VI semiconductors, are quasi-two-dimensional (2D) materials that exhibit unusual physical properties (high mobility of electric charge carriers, wide photoresponse bands, marked anisotropy of electrical, and optical properties, etc.). The GaSe and GaS, together with their solid solutions (GaS_x_Se_1−x_), are typical and outstanding representatives of this class of materials. 

The GaSe plates, kept for a long time in a normal atmosphere, are covered with a layer composed of gallium oxides [26]. The oxidation process of GaS and GaSe lamellae at high temperatures was studied in several works [27,28,29,30]. By conducting a heat treatment of GaS plates in argon flow at temperatures in the range of 700–900 °C, microsheets of *β*–Ga_2_O_3_ are formed on their surface [29]. Additionally, *β*–Ga_2_O_3_ nanowires and nanoribbons were obtained by high-temperature (≥900 °C) heat treatment of GaSe plates in argon/airflow [30,31].

Under certain technological conditions, *β*–Ga_2_O_3_/Ga_2_Se_3_ and *β*–Ga_2_O_3_/Ga_2_S_3_ nanocomposites can be obtained, which are prospective materials for expanding the application area of Beta–Gallium Oxide.

Depending on the arrangement of elementary Se(S)-Ga-Ga-Se(S) planar packings with respect to each other, four polytypes (*α*, *β*, *γ*, and *ε*) of GaSe single crystals were distinguished. The GaSe and GaS single crystals obtained by the Bridgman technique correspond to the *ε* and *β* phases, respectively. The layered compounds GaS and GaSe are known to form a continuous series of GaS_x_Se_1−x_ (0 ≤ x ≤ 1) solid solutions. In Refs. [32,33], appealing to X-ray diffraction (XRD) analysis and Raman spectroscopy, it was demonstrated that *ε* and *β* phases predominate in 0 ≤ x ≤ 0.01 and 0.5 ≤ x ≤ 1 composition, respectively, while the *γ* phase is characteristic for single crystals with the composition 0.05 ≤ x ≤ 0.40.

In this work, the chemical and elemental composition, surface morphology, light absorption in the region of the fundamental absorption edge, photoluminescence (PL), and photoconductivity of the layer formed by the heat treatment of single crystalline GaS_x_Se_1−x_ solid solutions in a water vapor-rich atmosphere (AVH_2_O) at a temperature of 900 °C are studied.

## 2. Materials and Methods

The samples under investigation are micro- and nanocomposite structures based on *β*–Ga_2_O_3_ on single crystal GaS_x_Se_1−x_ solid solutions (0 ≤ x ≤ 1) as substrates. Single crystals with x = 0, 0.17, 0.50, 0.80, 0.95, and 1 compositions were prepared by a modified Bridgmann-Stockbarger technique. The synthesis of compounds from chemical elements Ga (5N), Se (5N), and spectrally pure S, taken in stoichiometric amounts, and the preparation of single crystals were performed in a three-zone (zone I, zone II, and zone III) furnace. Initially, the furnace axis is tilted at ~30 degrees towards the horizontal, and the foil with Ga, S, and Se is placed in sectors I and II. The temperature in sector II was increased slowly at a rate of 100 °C/h, up to 1100 °C, while in sector I, it is maintained at the melting point of Se (S). As the amount of condensate at the outer, cold end of the ampoule decreases, the temperature in sector I increases at a rate of ~50 °C/h up to ~1200 °C. At this temperature, for 20 g of substance in the ampoule, the synthesis took about 6 h. Throughout the synthesis, the ampoule was subjected to mechanical vibrations with a frequency of ~50 Hz.

Thereafter, the melting furnace was moved vertically, its sector III set to ~700 °C, and the melt ampoule proceeded through the temperature gradient between sectors II and III, with a speed of ~1 mm/h. After the melt solidified, the temperature in the furnace was decreased at a rate of ~100 °C/h down to ambient temperature.

From as-synthesized bulk single crystal ingots, through mechanical splitting perpendicularly to the *C*_6_ axis, plane-parallel plates with thicknesses between 20 and 500 µm have been obtained. They were heat treated in an AVH_2_O, using a tube furnace with a MoSi_2_ heating element at temperatures from 850 to 920 °C for 1–6 h.

The structure, composition, and surface morphology of the samples under study were investigated by XRD, Energy Dispersive X-ray Spectroscopy (EDXS), and Raman spectroscopy, as well as Scanning Electron Microscopy (SEM).

The XRD patterns (CuK*α* radiation, *λ* = 1.5406 Å) of the samples were recorded in the 2*θ* angular range of 20–90° with an angular resolution of 0.02°, using a PANalytical Empyrean diffractometer, in Bragg-Brentano (*θ*–2*θ*) geometry. Raman spectroscopy measurements were performed at room temperature in the backscatter configuration (180 deg), with a WITec alpha300 R spectrometer using an Ar^+^ ion laser (wavelength *λ* = 480 nm and power of ~2 mW) as excitation light source. The spectral frequency resolution of measurements was ~2 cm^−1^.

The morphology of the samples surface was examined by SEM-EDX, using a Zeiss Ultra Plus electron microscope (7 kV, 10 μA) equipped with an EDX analysis system [a Si/Li detector (Noran, Vantage System)]. Diffuse reflectance spectra of the surface of micro- and nanostructured *β*–Ga_2_O_3_ layer on single crystal substrate (GaS, GaSe, and GaS_x_Se_1−x_ solid solutions) have been recorded with a Specord M-40 spectrophotometer with a spectral energy resolution of 0.5 meV, equipped with accessories for diffuse reflectance measurements at an angle of 90° to the incident beam.

Photoluminescence spectra were recorded with a specialized photometric setup which included a high-power MDR-2 monochromator with diffraction gratings (600 and 1200 mm^−1^), equipped with a photomultiplier with multi-alkali[(Na_2_K)Sb + Cs] photocathode with a quartz window. A 200 W argon vapor lamp (UV-C light source) and a DRS-500 filtered mercury lamp (wavelength *λ* = 546 nm) were used as excitation sources of photoluminescence and photoconductivity of the samples under study. The photocurrent in the circuit was recorded with a V7-30 microvoltmeter-electrometer.

## 3. Results and Discussion

### 3.1. Structural Studies

**Figure 1:**
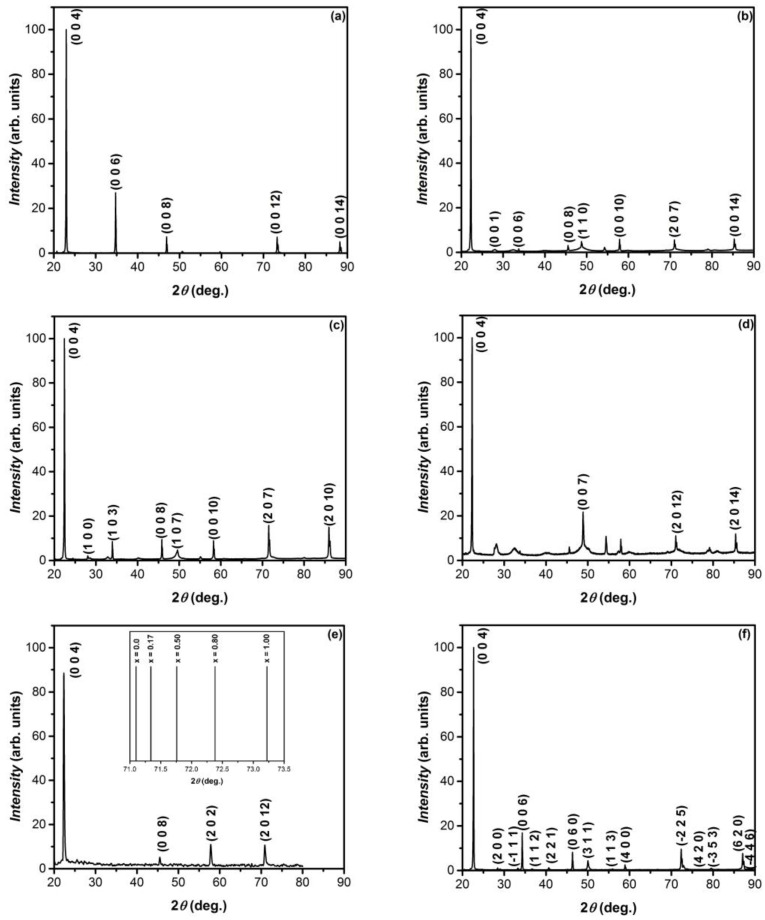
XRD patterns of single crystal GaS_x_Se_1−x_ solid solutions obtained by Bridgman-Stockbarger technique: (**a**) *x* = 1; (**b**) *x* = 0.80; (**c**) *x* = 0.50; (**d**) *x* = 0.17; (**e**) *x* = 0.00; and (**f**) composite obtained by 6 h heat treatment of GaS_0_._8_Se_0_._2_ single crystals in AVH_2_O, at 800 °C. Inset of (**e**): 2*θ* angular displacement of the (0012) line, as a function of the composition (x) of GaS_x_Se_1−x_ solid solutions.

The crystal structure and phase purity of GaS and GaSe single crystals and related solid solutions were investigated using X-ray diffractograms, recorded in the 2θ angular range between 20 and 90°. Figure 1a shows the XRD diagram of GaS, in which the intense lines positioned at 2θ angles of 22.985°, 34.781°, 46.840°, and 73.928° are emphasized; they correspond to (004), (006), (008), and (0012) crystal planes, respectively, parallel to the *C*_6_ axis.

According to (ICDD-JCPDS) PDF 840499 card, these XRD peaks can be identified as diffraction lines of *β*–GaS polytype with lattice constants *a* = 3.592 Å and *c* = 15.465 Å. In the XRD diagram of GaSe single crystals obtained by the Bridgman technique, in the 2*θ* range from 20 to 90°, four peaks are clearly emphasized [Figure 1e]. The highest intensity lines can be ascribed to (002), (004), (008), and (0012) crystal planes parallel to the *C*_6_ axis. According to PDF card no. 00-037-0931, these peaks correspond to the hexagonal ε–GaSe phase with crystal lattice constants *a* = 3.749 Å and *c* = 15.907 Å.

The ionic radius of selenium (Se^6+^) is 1.45 times greater than that of sulfur (S^6+^). Consequently, selenium substitution by sulfur results in crystal lattice deformation of solid solutions. This process obviously leads to the change of the arrangement of S/Se-Ga-Ga-S/Se elementary packings with respect to each other in the unit cell, therefore also to the polytypism of respective crystal structures. As demonstrated by XRD measurements in [32], the constant *C* increases monotonically with the composition (x) of a single crystalline solid solution in the ranges of 0.0 ≤ x ≤ 0.3 and 0.4 ≤ x ≤ 1.

Figure 1b shows the XRD pattern of GaS_x_Se_1−x_ solid solution crystals with the composition x = 0.80. According to Serizawa and coworkers [33], single crystals with this composition correspond to the *γ*–GaSe polytype. The diffraction lines centered at 2*θ* angles equal to 22.30°, 28.09°, 33.76°, 45.58°, 48.78°, 54.25°, 57.92°, and 71.02° are in good correlation with the characteristic reflections of *γ*–GaSe single crystals (PDF card no. 811974). The small increase in the values of 2*θ* angles corresponds to the diffraction on atomic planes of the solid solution for the compositions x = 0.80 and x = 0 (pure GaSe). It is determined by the contraction of the crystal lattice caused by the replacement of the Se ions with S ions (the ionic radius of S^6+^ is ~1.45 times smaller than that of Se^6+^). Further contraction of the crystal lattice of GaS_x_Se_1−x_ compound occurs together with increasing x (Figure 1d,e).

In Figure 1e (inset), the variation of 2*θ* diffraction angle on the (0012) atomic planes is schematically presented, from which the increase of 2*θ* values together with the composition (x) of the GaS_x_Se_1−x_ solid solution can be clearly ascertained.

**Figure 2:**
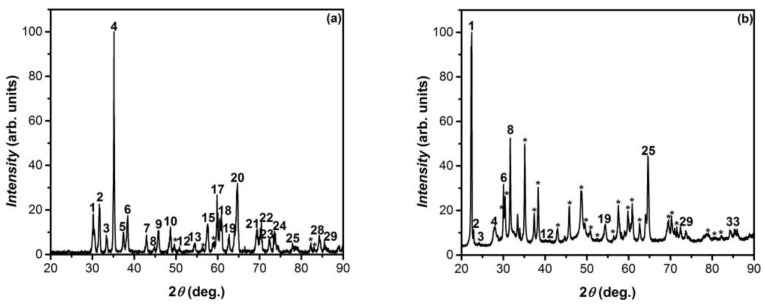

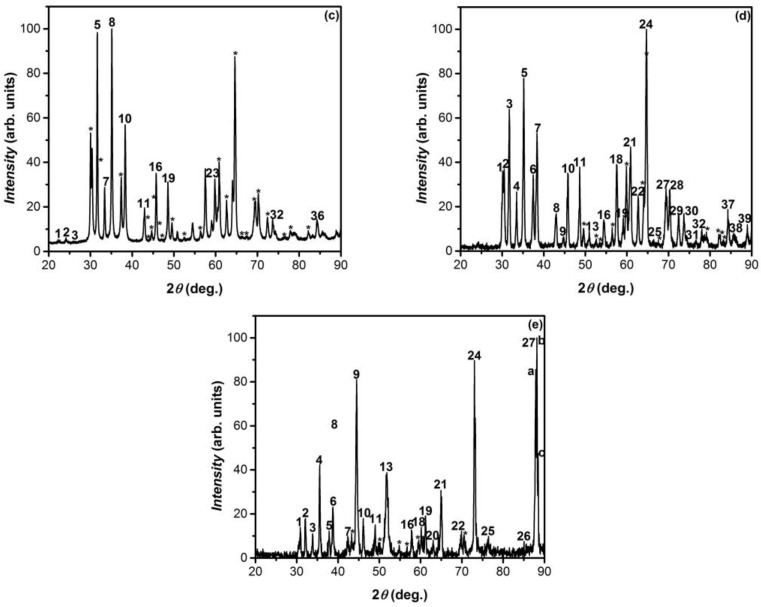
XRD diagrams of the composite obtained by heat treatment in AVH_2_O, at 900 °C for 6 h, of single crystal GaS_x_Se_1−x_ plates for (**a**) x = 0.00; (**b**) x = 0.17; (**c**) x = 0.50; (**d**) x = 0.80; (**e**) x = 1. The asterisks represent the numbers assigned to the diffraction peaks (omitted for space saving reasons).

The XRD patterns, in the 2*θ* range of 20–90°, of the material obtained by the heat treatment (at 900 °C, for 6 h) of GaSe and GaS single crystals in AVH_2_O are shown in Figure 2a–e, respectively.

By comparing these two figures, one can clearly ascertain the good correlation between XRD peaks which, according to the PDF card 741-776, can be identified as diffraction lines corresponding to *β–Ga_2_O_3_* single crystals with the cell parameters: *a* = 12.23 Å, *b* = 3.04 Å, *c* = 5.80 Å, and *β* = 103.7°. 

Using Raman spectroscopy analysis, in [27], it was demonstrated that by oxidizing the GaSe single crystal, a composite of Ga_2_O_3_ crystallites and amorphous Se is obtained. At the oxidation temperature of 800–950 °C, the *β*–*Ga*_2_*O*_3_ layer on the surface of the GaS/GaSe single crystals sublimes, forming *β*–*Ga*_2_*O*_3_ vapors on the sample surface. In [30], it is shown that *β*–*Ga*_2_*O*_3_ nanoformations (nanowires, nanoribbons, nanotowers, etc.) are formed by vapor condensation on the surface of the sample.

The contour of X-ray diffraction lines from nanowires/nanoribbons broadens together with decreasing nanometer crystallite sizes. The average sizes of the crystallites can be approximated using the Debye-Sherrer formula [33]:

(1) $$d=\frac{k\lambda }{{\beta cos\theta }_{hkl}}$$

where *k* is the Sherrer constant, equal to 0.94, *λ* is X-ray wavelength (1.54060 Å), *θ_hkl_* denotes the Bragg diffraction angle for the family of lattice planes with Miller indices (*hkl*), and *β* represents the angular full width at half maximum (FWHM) intensity of the (*hkl*) peak. The average sizes of the *β*–*Ga*_2_*O*_3_ crystallites formed on the surface of GaSe and GaS plates have been estimated using Equation (1), considering the diffraction lines at 2*θ* = 57.56 and 57.85° and were found to be equal to 19 and 20 nm, respectively.

Figure 1e,f show the XRD patterns of GaS_x_Se_1−x_ solid solution with x = 0.80 before and after a 6 h heat treatment in AVH_2_O at 850 °C, respectively. Thus, it can be seen that in the last diagram (Figure 1f) the diffraction peaks characteristic of the primary composite are dominant, while the presence of *β*–Ga_2_O_3_ is manifested by a trace of the diffraction line at 2*θ* = 33.3°. Lower intensity peaks located at (2*θ* angles of) 28.4, 33.3, 36.7, 46.8, 58.9, 72.0, and 79.5° can be identified (PDF card no. 76-2310) as reflections characteristic of Ga_2_Se_3_ crystallites-monoclinic crystal lattice with the parameters *a* = 6.60 Å; *b* = 6.660 Å; *c* = 11.650 Å; and *γ* = 108.72°. The lines with maxima at 34.3 and 40.4° correspond to Ga crystallites-trigonal lattice with the parameters *a* = *b* = 9.087 Å; *c* = 17,020 Å; and *γ* = 120° (PDF card no. 01-071-0505). When the heat treatment temperature is increased by 50°, the surface of GaS and GaSe lamellae and GaS_x_Se_1−x_ solid solutions (0 ≤ x ≤ 1) is covered with a nanostructured layer of gallium oxide.

**Figure 3:**
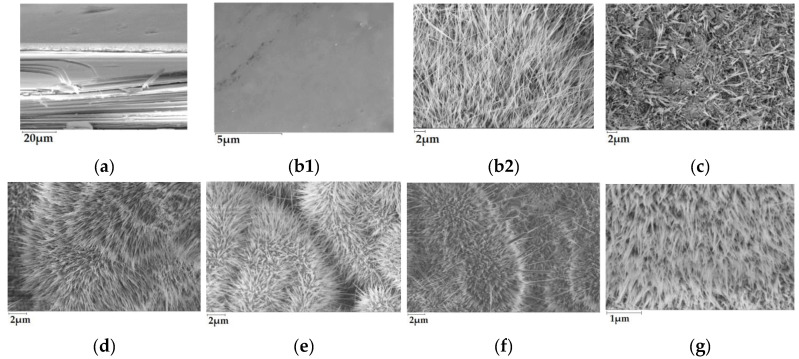
SEM images of the (**a**) surface of the section parallel to the *C*_6_ crystallographic axis of GaS_x_Se_1−x_ (x = 0.85) single crystals, heat treated in AVH_2_O at 850 °C for 6 h; (**b1**) (0001) surface of GaS lamella, heat treated in AVH_2_O at 750 °C and (**b2**) 900 °C for 6 h; surface of GaS_x_Se_1−x_ single crystals, heat treated in AVH_2_O at 900 °C for 6 h, with compositions (**c**) x = 0.05; (**d**) x = 0.17; (**e**) x = 0.50; (**f**) x = 0.80; (**g**) x = 0.00.

Figure 3 shows the cross-sectional SEM micrograph of GaS_x_Se_1−x_ solid solution layer with the composition x = 0.85, subjected to a heat treatment in AVH_2_O at 850 °C for 6 h, from which, together with single crystal plane-parallel lamellae, the *β*–*Ga*_2_*O*_3_ oxide layer formed on the III-VI (0001) surface is clearly emphasized. 

Figure 1d and Figure 2b show the XRD diagrams of the primary and oxidized (in AVH_2_O, at a temperature of 900 °C for 6 h) GaS_0_._17_Se_0_._83_ single crystals, respectively. The diffraction lines in Figure 1b, according to PDF card no. 01-078-1927, correspond to the GaSe hexagonal lattice-*ε* polytype [33]. In the diagram in Figure 2b, together with the XRD lines of the primary material, intense peaks with maxima positioned at 2*θ* angles of 15.60, 18.90, 30.00, 31.70, 35.00, 54.40, 59.80, 60.80, and 64.60° are shown, which according to PDF card no. 431012, correspond to *β*–*Ga*_2_*O*_3_ polytype (monoclinic lattice) with parameters *a* = 12.23 Å, *b* = 3.04 Å, *c* = 5.80 Å, and *β* = 103.7°. At the same time, this diagram shows three diffraction peaks with maxima located at (2*θ* angles of) 28.00, 38.30, and 62.64°, which according to PDF card no. 76-0975, can be ascribed to Ga_2_Se_3_-monoclinic lattice with parameters *a* = 6.660, *b* = 11.650, *c* = 6.649 Å, and *β* = 108.84°.

The average crystallite sizes of Ga_2_Se_3_ and Ga_2_O_3_ were determined using Formula (1) from the broadening of diffraction peaks located at 28.33 and 54.40°, respectively, and were found to be equal to 10 nm and 17 nm, respectively.

The XRD patterns of GaS_x_Se_1−x_ single crystals (solid solutions with the compositions *x* = 0.5 and 0.8), primary and after heat treatment at 900 °C for 6 h, are presented in Figure 1c,b and Figure 2c,d, respectively. The peaks of X-ray diffractograms in Figure 2c,d, according to PDF card no. 431-1012, can be interpreted as diffraction lines from the sets of crystal planes of monoclinic *β*–Ga_2_O_3_, with lattice parameters *a* = 12.23 Å, *b* = 3.04 Å, *c* = 5.80 Å, and *β* = 103.79°. The absence of the intense line at 2*θ* = 22.40° (Figure 2d) indicates complete oxidation of the sample.

### 3.2. Morphological and Compositional Analysis

The SEM images of the surface of the *β*–Ga_2_O_3_ layer, obtained by a heat treatment in a normal atmosphere at 850/900 °C for 6 h, of single crystalline GaS, GaSe, and GaS_x_Se_1−x_ lamellae with the compositions x = 0.05, 0.17, 0.50, and 0.85, are shown in Figure 3b–g.

As can be seen in Figure 3(b1), by a heat treatment at 750 °C in AVH_2_O, a smooth layer of white material is formed on the (0001) surface of GaS lamella.

In GaS and GaSe single crystals, the valence bonds at the (0001) surface of elementary S/Se-Ga-Ga-S/Se stratified packings are practically closed. Therefore, the surface defects of elementary III-VI packings can constitute, most likely, nucleation germs of the *β*–Ga_2_O_3_ layer. The defect density on the (0001) surface of GaSe plates is of the order of 10^10^–10^12^ cm^−2^ [26]. On the other hand, the valence bonds on the edge of GaS/GaSe plates are open, which facilitates the formation of the *β*–Ga_2_O_3_ layer on the edge. Under normal atmospheric conditions, the oxidation process of GaSe plates is long-lasting [26], and at temperatures over 750 °C, edge oxidation [(1000) surface] is especially favored.

The formation mechanisms of both *β*–Ga_2_O_3_ and related nanoformations through the heat treatment of gallium chalcogenides in an oxygen-enriched atmosphere have been studied in Refs. [28,29,34,35,36,37]. Formation reactions of gallium oxide (*β*–Ga_2_O_3_) by high-temperature heat treatment of GaSe lamellae in an Ar/O_2_ atmosphere can proceed with the formation of Ga_2_Se_3_ compound and/or through direct transformation, as follows [35,37]:

(2) $$3GaSe+\frac{3}{2}\;3GaSe+\frac{3}{4}O_{2}\to {Ga}_{2}{Se}_{3}+\frac{1}{2}{Ga}_{2}O_{3,}$$

(3) $${Ga}_{2}{Se}_{3}+\frac{3}{2}O_{2}\to {Ga}_{2}O_{3}+3Se,$$

(4) $$GaSe+\frac{3}{4}O_{2}\to \frac{1}{2}{Ga}_{2}O_{3}+Se.$$

Analogously, *β*–Ga_2_O_3_ compound is formed by the heat treatment of single crystalline GaS lamellae [29] in AVH_2_O. In addition, the process of obtaining *β*–Ga_2_O_3_ oxide by photo-oxidation of GaSe single crystals is influenced by the amount of water vapor in the atmosphere [37,38]. In mentioned works, the photo-oxidation reaction takes place in two stages through the reactions:

(5) $$3GaSe+\frac{3}{2}HO_{2}+\frac{9}{4}O_{2}\to Ga({OH)}_{3}+{Ga}_{2}O_{3}+3Se,$$

(6) $$2Ga({OH)}_{3}\to {Ga}_{2}O_{3}+3H_{2}O.$$

Figure 3c shows a typical SEM image of the nanoformations formed on the (0001) *ε*–GaSe surface of the plates subjected to heat treatment at 900 °C for 6 h in AVH_2_O. The nanoformations display chaotically oriented needles with micrometric-scale lengths of (1–5) µm.

As can be seen from the SEM image (Figure 3g), the *β*–Ga_2_O_3_ layer obtained by a 6 h heat treatment of GaS_x_Se_1−x_ (x = 0.17) solid solution in AVH_2_O at 900 °C is formed by nanowires with lengths of 5–7 µm. In addition to *β*–Ga_2_O_3_, the composition of this material is likely to include an O_2_ surplus of ~6% (Table 1).

By heat treatment (at 900 °C, for 6 h in AVH_2_O) of GaS_0.5_Se_0.5_ and GaS_0.8_Se_0.2_ solid solutions (Figure 3e,f), a homogeneous assembly of *β*–Ga_2_O_3_ nanowires is obtained. As can be seen from this image, the surface of the primary material plate undergoes major deformations and is covered with nanowire assemblies, the length of which increases together with the duration of the heat treatment. These materials are probably composed of *β*–Ga_2_O_3_ nanowires with a surplus of ~1.2% and 0.5% atomic oxygen, respectively. Therefore, it can be observed that with the increase in the sulfur concentration x of the primary solid solution, the excess oxygen concentration in the layer of *β*–Ga_2_O_3_ nanoformations increases.

The elemental composition of the layer penetrated by the 20 keV electron beam was determined from the EDXS spectra. The thickness of the *β*–Ga_2_O_3_ layer (*r*) penetrated by the electron beam can be approximated using the Kanaya-Okayama empirical formula [39]:

(7) $$r=\frac{0.0276}{\rho }\cdot \frac{A}{Z^{\sigma }}E_{0}^{\gamma },$$

where *A* is the mass number, *Z* denotes the atomic number, *ρ* is the density (0.89 g/cm^3^), and *E*_0_-the electron energy, in keV; for *E*_0_ > 5 keV, the power coefficient is *γ* = 1.67. For *E*_0_ = 2 keV, *ρ* = 5.88 g/cm^3^, *A* = 69.7, *Z* = 31, it results in the penetration depth of the electron beam in EDXS measurements of *β*–Ga_2_O_3_ being ~2.2 µm.

**Figure 4:**
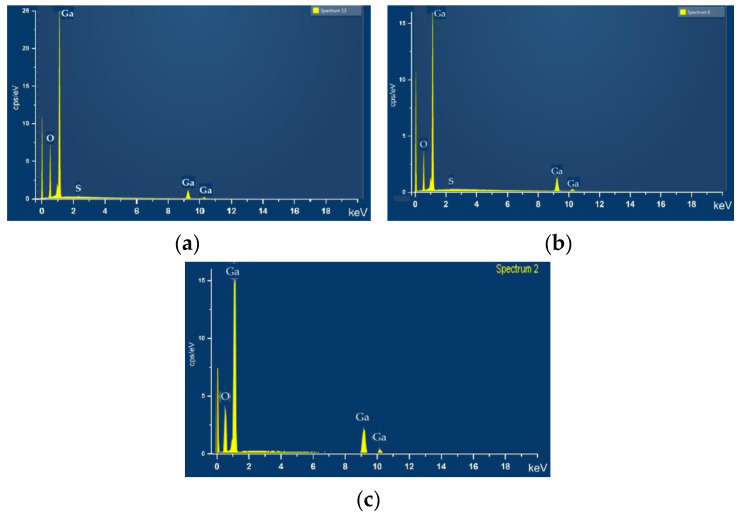
EDX spectra of *β*–Ga_2_O_3_ layer formed on the (0001) surface of single crystalline plates of GaS_x_Se_1−x_ solid solutions with the composition (**a**) x = 1, heat treated at 850 °C for 6 h in AVH_2_O; (**b**) x = 1, heat treated at 900 °C for 30 min; (**c**) x = 0.17, heat treated at 900 °C for 6 h in AVH_2_O.

The EDXS spectrum of *β*–Ga_2_O_3_ layer on the GaS substrate is shown in Figure 4, from which it can be seen that the penetrated layer contains 59.33% oxygen, 40.43% gallium, and 0.23% sulfur. Consequently, the O/Ga atomic ratio in this layer is 3/2, which corresponds to the stoichiometric compound Ga_2_O_3_. 

As the heat treatment temperature in AVH_2_O increases from 750 to 900 °C, a homogeneous layer of nanoribbons and nanowires is formed on the surface of the GaS lamellae (Figure 3(b1)). A field of *β*–Ga_2_O_3_ nanowires with lengths of tens of micrometers is formed on the surface of GaS plate subjected to the heat treatment at 900 °C for 30 min (Figure 3(b2)). The elemental composition of the nanowires/nanolamellae structures was determined from the EDXS spectra (Figure 4a,b). If we accept that following the thermal treatment of GaS plates in AVH_2_O at temperatures of 850 and 900 °C, the compound *β*–Ga_2_O_3_ is formed, then a surplus of gallium should result in the material obtained at 900 °C, which is emphasized in Table 1. This surplus of gallium, equal to ~2%, in the *β*–Ga_2_O_3_ layer formed on the surface of the GaS plate at a temperature of 850 °C, is increasing to ~10% in the nanowire layer formed at 900 °C and may be caused by different formation times of metallic Ga and *β*–Ga_2_O_3_.

Concentrations (in at. %) of oxygen (*C_O_*) and gallium (*C_Ga_*) in the *β*–Ga_2_O_3_ layers obtained by the heat treatment (0.5 and 6 h at 900 °C in AVH_2_O) of single crystal plates of GaS_x_Se_1−x_ solid solutions with the compositions x = 1, x = 0.80, x = 0.50, x = 0.17, and x = 0.05 are presented in Table 1. As can be seen from this table, with a 30 min heat treatment of GaS plates at 900 °C, a deficit of oxygen can be found in the as-formed *β*–Ga_2_O_3_ layer, while in the *β*–Ga_2_O_3_ layer formed on the solid solutions with x = 0.80 and x = 0.50, a surplus of oxygen is attested. The highest oxygen deficit of 11% is found in the *β*–Ga_2_O_3_ layer formed on the surface of *γ*–GaS_0_._17_Se_0_._83_ polytype.

**Figure 5:**
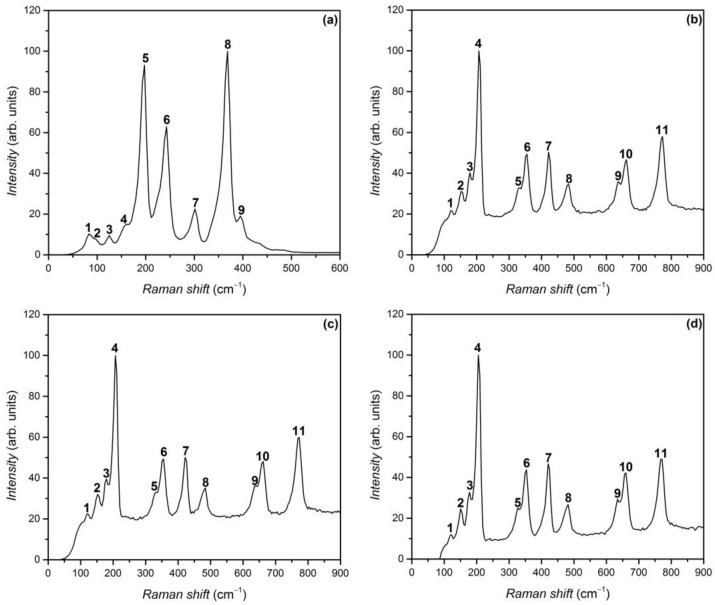
Raman spectrum, at 293 K, for (**a**) GaS/Ga_2_O_3_ composite, obtained by heat treatment of single crystalline GaS plates in AVH_2_O, at 750 °C for 6 h; (**b**) *β*–Ga_2_O_3_ crystallites from the composite material obtained by heat treatment of single crystalline *β*–GaS plates in AVH_2_O, at a temperature of 850 °C for 6 h; (**c**) 900 °C, for 30 min; and (**d**) 900 °C, for 6 h.

Raman spectra in the wavenumber range of 60–800 cm^−1^ of the material obtained by the heat treatment of GaS single crystals in AVH_2_O at temperatures of 750 and 900 °C are shown in Figure 5a and b, respectively. The vibrational spectrum in the spectral range of 100–400 cm^−1^ of the samples obtained at 750 °C contains three intense bands with maxima located at 189, 234, and 360 cm^−1^, together with five low-intensity bands at 74, 115, 149, 295, and 389 cm^−1^. The peaks positioned at 189, 295, and 360 cm^−1^ are identified in Refs. [40,41] as vibration modes of the hexagonal GaS lattice. Raman bands peaked at 74, 284, and 389 cm^−1^ corresponding to the vibration of the wurtzite-type α–Ga_2_S_3_ lattice [42]. The presence of a monoclinic *β*–Ga_2_O_3_ crystal phase in the layer under study is demonstrated by vibration bands with maxima at 115 and 149 cm^−1^. As demonstrated in [43,44], these bands are characteristic of the vibration modes of the monoclinic *β*–Ga_2_O_3_ lattice from the Raman spectra of *β*–Ga_2_O_3_ thin films and nanowire layers. The vibrational spectra of the layers composed of *β*–Ga_2_O_3_ nanoformations, formed by the heat treatment of GaS plates in the air at a temperature of 850 °C for 6 h and at 900 °C for 30 min, are near-identical (Figure 5b,c). In these spectra, the bands with maxima located at 114, 146, 171, 200, 327, 349, 416, 478, 630, 656, and 767 cm^−1^ are clearly emphasized. This structure is also characteristic of the Raman spectra of layers composed of *β*–Ga_2_O_3_ nanoformations, obtained by thermal treatment (in AVH_2_O, at 900 °C for 6 h) of GaS_x_Se_1−x_ solid solutions with the compositions x = 0.17, 0.50, and 0.80. The Raman frequencies, intensities, and symmetry of the vibration modes of *β*–Ga_2_O_3_ layer obtained by the heat treatment in AVH_2_O of single crystal plates of GaS_x_Se_1−x_ solid solutions with x = 0.00, 0.17, 0.50, 0.80, and 1, are included in Table 2.

In this table, the symmetry of the vibration modes according to Ref. [5] was also included. One can conclude that by the heat treatment of GaS_x_Se_1−x_ (0 ≤ *x* ≤ 1) solid solution single crystals in AVH_2_O at temperatures of 850 and 900 °C with a duration from 30 min to 6 h, layers composed of *β*–Ga_2_O_3_ nanowires and nanoribbons are formed on the lamellae surface.

### 3.3. Optical and Photoluminescence Properties

The absorption threshold of micro- and nanostructured *β*–Ga_2_O_3_ layers, obtained by high-temperature heat treatment in AVH_2_O of single crystalline GaS_x_Se_1−x_ solid solution lamellae, was studied from diffuse reflectance (*R_d_*) measurements as a function of wavelength using the Kubelka-Munk function [45]:

(8) $$F\left(R_{d}\right)\frac{{\left(1-R_{d}\right)}^{2}}{{2R}_{d}}=\frac{\alpha }{S},$$

where *R_d_* = (*I*/*I*_0_)_dif_ (with I and I_0_ denoting the intensities of diffuse reflected radiation by sample surface and BaSO_4_ powder standard), *α* is the diffusion coefficient (usually expressed in cm^−1^), and *S* (in cm^−1^) represents the diffusion coefficient–a wavelength-independent parameter for particles with average sizes greater than the incident wavelengths.

As clearly revealed by the SEM images of *β*–Ga_2_O_3_ layers, their surface consists of micrometer-sized grains covered with nanoformations. It is considered that inhomogeneities with micrometric sizes behave as diffuse scattering centers of incident light, so one can admit that *F*(*R_d_*) ~ *α*.

In the case of parabolic energy bands, the band gap (*E_g_*) and the absorption coefficient (*α*) of the semiconductor material are related by Equation (9) [46]:

(9) $$\alpha h\nu =A{\left(h\nu -Eg\right)}^{n},$$

where *A* is a characteristic parameter for respective transitions, direct (*n* = 1/2) or indirect (*n* = 2), which is independent of photon energy, *hν*.

Taking into account Equations (8) and (9), the relation between the function *F*(*R_d_*) and the direct band gap energy *E_gd_* can be expressed by:

(10) $${\left[F\left(R_{d}\right)h\nu \right]}^{2}=B\left(h\nu -{Eg}_{d}\right),$$

with *B* denoting a new constant, also independent of photon energy.

**Figure 6:**
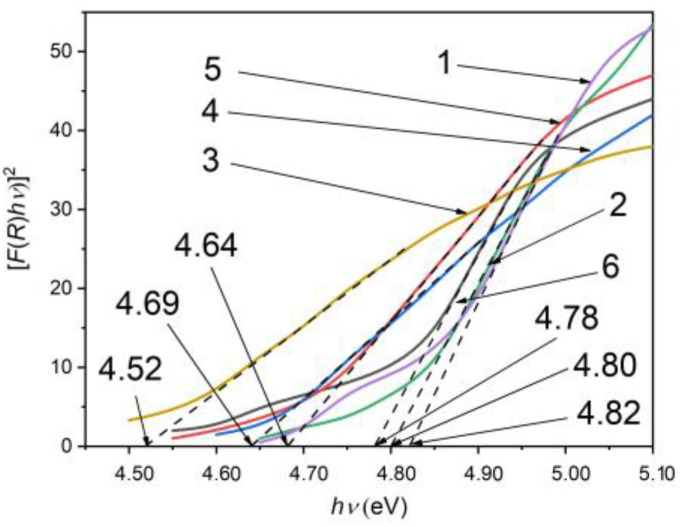
Determining the direct band gap of *β*–Ga_2_O_3_ layer on substrate of GaS_x_Se_1−x_ solid solution with compositions *x* = 1 (1), 0.95 (2), 0.50 (3), 0.17 (4), 0.05 (5), 0.00 (6).

Figure 6 shows the [*F*(*R_d_*)*hν*]^2^ dependencies on the photon energy, from which the direct band gap of the *β*–Ga_2_O_3_ layer obtained by the heat treatment of single crystalline GaS_x_Se_1−x_ lamellae with *x* = 1.00, 0.95, 0.50, 0.17, 0.05, and 0.00, in AVH_2_O at 900 °C, was found to equal 4.82, 4.80, 4.52, 4.64, 4.69, and 4.78 eV, respectively. Therefore, the band gap of the nanostructured *β*–Ga_2_O_3_ layer depends on the composition of GaS_x_Se_1−x_ solid solution and decreases with the increase in the GaSe concentration in GaS. The lowest value, 4.52 eV, is observed in the *β*–Ga_2_O_3_ layer formed on the material at the frontier between the *γ* and *β* polytypes.

The band gap of nanostructured *β*–Ga_2_O_3_ layers obtained by calcination at temperatures of 870–900 °C in an AVH_2_O, determined from the spectral reflectance analysis, varies from 4.50 eV to 4.70 eV [47,48]. As demonstrated in Refs. [49,50], the forbidden bandwidth, *E_g_*, is influenced by the growth technology of *β*–Ga_2_O_3_. The band gap of *β*–Ga_2_O_3_ layers obtained by Ar/O_2_ flow transport of Ga vapors is 4.83 eV [51]. The optical band gap of *β*–Ga_2_O_3_ layers prepared by the PLD technique on a heated support varies from 5.09 eV to 5.11 eV [52]. In the case of *β*–Ga_2_O_3_ single crystals, *E_g_* is equal to 4.68 eV and is weakly influenced by the crystallographic direction of the UV radiation flux [53].

**Figure 7:**
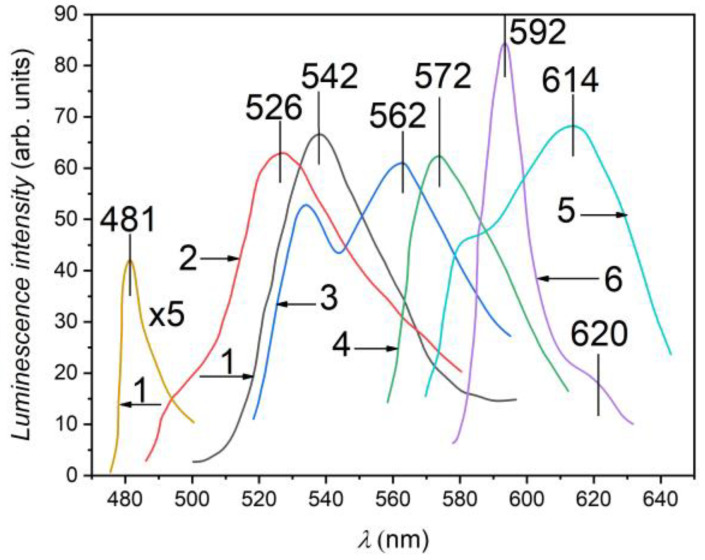
Photoluminescence of GaS_x_Se_1−x_ single crystals at 80 K upon excitation with monochromatic radiation (*λ* = 405 nm, *P* = 15 mW/cm^2^). (1) x = 1; (2) x = 0.95; (3) x = 0.80; (4) x = 0.50; (5) x = 0.17; and (6) x = 0.00.

In Figure 7, the PL spectra of *β*–Ga_2_O_3_ films prepared by heat treatment (at 900 °C for 6 h) in AVH_2_O of single crystalline GaS (1) and GaSe (6), as well as of GaS_x_Se_1−x_ (x = 0.17, 0.50, 0.80, and 0.95) solid solution lamellae at the temperature 80 K. Photoluminescence was excited using a 405 nm wavelength radiation.

Gallium sulfide (GaS) is known as an indirect semiconductor with a band gap of 2.57 eV. Also, the direct band gap in GaSe is 1.90 eV. In addition, the *E_g_* value in GaS_x_Se_1−x_ (0 ≤ x ≤ 1) solid solutions lies in the range between 1.90 and 2.57 eV [54]. The PL spectrum of *β*–GaS crystals at 80 K is composed of two bands, one in the green spectral region and the other in the yellow region.

The PL bands with weakly asymmetric contour, located near the fundamental absorption edge of GaS and GaSe single crystals, peaked at 481 nm (2.578 eV) and 592 nm (2.094 eV) and are well known in the literature. In Refs. [55,56], they are interpreted as radiative emission of indirect excitons located in GaS and of direct excitons located in GaSe crystals (Figure 7, curves 1 and 6), respectively. The energy position of the particularity by 620 nm (2.00 eV) (curve 6) coincides with the energy of indirect excitons in GaSe. The band with a maximum at 542 nm predominates in the PL spectrum of undoped GaS crystals and is of an impurity nature. The PL spectra of the crystals with the compositions x = 0.95, 080, and 0.17 consist of broad, asymmetric bands with maxima positioned at 526 nm (2.36 eV), 572 nm (2.17 eV), and 614 nm (2.02 eV), respectively. These bands, together with the band with a maximum at 562 nm from the PL spectrum of the GaS_x_Se_1−x_ crystals with the composition x = 0.80, are located in the optical transparency region of respective crystals and are probably of an impurity nature. In Ref. [57], the absorption and PL spectra at room temperature of 2D GaS_x_Se_1−x_ solid solutions are studied, from which a good correlation was attested between the PL peak positions and the indirect band gap determined from the diffuse reflectance spectra.

**Figure 8:**
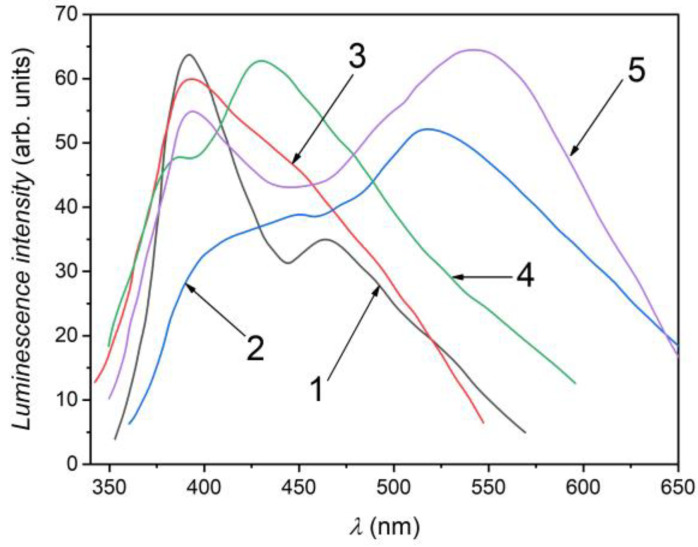
Photoluminescence of nanostructured *β*–Ga_2_O_3_ layer on GaS_x_Se_1−x_ substrate with compositions (1) x = 0.00, (2) x = 0.17, (3) x = 0.50, (4) x = 0.80, (5) x = 1.

Figure 8 shows the PL spectra at 80 K of the *β*–Ga_2_O_3_ layers formed by a 6 h heat treatment at 900 °C in AVH_2_O of single crystalline GaS (1) and GaSe (6) lamellae, as well as those of GaS_x_Se_1−x_ solid solutions with x = 0.17, 0.50, 0.80, and 0.05.

The PL spectra of the *β*–Ga_2_O_3_ layer on GaS_x_Se_1−x_ solid solution substrate was excited with the radiation of an emission band with a maximum at 254 nm and a half-width of 15 nm selected from the spectrum of a ~400 W Ar-arc lamp using a SiO_2_ prism monochromator that was intensity-modulated at 95 Hz. The PL spectra of *β*–Ga_2_O_3_ layers on GaS and GaSe plates, as well as on GaS_x_Se_1−x_ (0 < x < 1) solid solution crystals, contain two bands: a narrow one with the maximum at the UV-violet frontier and another one with a broad contour, displaying peak intensity in the green-red region. The UV-violet PL band is present in both *β*–Ga_2_O_3_ thin films and nanostructured layers [54,58,59]. A PL band with a maximum of 390 nm was also observed in the cathodoluminescence spectrum of an assembly of nanowires obtained by the CVD technique [60]. In Ref. [59], it was observed that the structure of the PL spectrum of the nanostructured *β*–Ga_2_O_3_ depends on the excitation wavelength. If for an excitation wavelength of 254 nm, the PL spectrum of the assembly of *β*–Ga_2_O_3_ nanoribbons is composed of two poorly outlined bands with maxima positioned at 375 nm and 414 nm, then upon a 325 nm excitation, in the emission spectrum, two intense bands with maxima at 455 and 528 nm are emphasized.

In the PL spectrum (at the temperature 80 K) of the nanostructured *β*–Ga_2_O_3_ layer formed on the surface of GaS lamella (Figure 8, curve 5), two bands with maxima positioned at 395 nm (3.14 eV) and 549 nm (2.28 eV) are clearly visible. The PL band peaked at the wavelength of 395 nm is well studied in [60,61,62]. In mentioned papers, it is assumed that this band is formed as a result of electron-hole recombination in the pair vacancy (V_O_–V_Ga_) defect. In Refs. [63,64], it is admitted that oxygen defects (V_O_) form donor levels in the energy range of 0.60–2.16 eV, while gallium vacancies (V_Ga_) form two acceptor levels, located at ~0.19 eV and 0.62 eV with respect to the valence band top.

The forbidden band gap of the nanostructured *β*–Ga_2_O_3_ layer on the GaS substrate is equal to 4.82 eV (Figure 6, curve 1). The energy diagram (deep levels) of *β*–Ga_2_O_3_ crystals was established from Deep-Level Transient Spectroscopy (DLTS) and Deep Level Optical Spectroscopy (DLOS) analyzed in [63,64]. According to them, the emission band with a maximum at 395 nm can be interpreted as donor-acceptor (DA) radiative emission in the vacancy pair (V_O_, V_Ga_) by recombination of electrons from the donor level with energy of 0.80 eV (relative to the conduction band minimum), with the holes on the acceptor level with energy *E_c_* = 3.94 eV (with respect to the valence band maximum). Additionally, the PL band with a maximum of 549 nm can be interpreted as the recombination of electrons from the deep level with an energy of 1.66 eV and holes from the acceptor level located at ~0.88 eV above the valence band top.

The PL spectrum of the *β*–Ga_2_O_3_ layer on the GaSe substrate is composed of two bands with maxima located at 390 nm (3.18 eV) and 460 nm (2.70 eV) wavelengths. The violet emission band in the *β*–Ga_2_O_3_ layers on GaS and GaSe substrates probably has the same nature and is determined by the presence of oxygen and gallium vacancies. An emission band with a maximum of 460 nm was observed in the PL spectrum of the nanostructured *β*–Ga_2_O_3_ layer (nanoribbons and nanowires) obtained by a heat treatment at 950 °C of gallium in water vapor. This band is interpreted in [65] as electron-hole recombination in the donor-acceptor pair. As seen in Figure 8, curve 2, the band at 460 nm predominates and can be interpreted, based on the energy level diagram of *β*–Ga_2_O_3_ compound [63,64], as the radiative transition of electrons from the conduction band on the energy level located at 2.71 eV below the conduction band minimum. We note that the PL spectrum of the *β*–Ga_2_O_3_ powders studied in the Refs. [66,67] consists of a broad band with a poorly outlined maximum at 476 nm (2.60 eV). The structure of the PL spectrum of nanostructured *β*–Ga_2_O_3_ depends on several (technological) factors (nature of the material, synthesis method, and temperature, etc.). As demonstrated in [68], the presence of a broad and poorly emphasized emission maximum at wavelengths of 510–530 nm is characteristic of the luminescence of *β*–Ga_2_O_3_ nanoparticles.

In the PL spectra, at 80 K, of the *β*–Ga_2_O_3_ layer formed on the substrate of GaS_x_Se_1−x_ (x = 0.17) solid solutions, the violet band with peak intensity in the wavelength range of 380–410 nm, characteristic for the PL spectrum of the nanostructured *β*–Ga_2_O_3_, and the green band with maximum at 540–560 nm are well marked. At the same time, in the PL spectrum of the layer formed by the heat treatment in AVH_2_O at 900 °C of GaS_0_._17_Se_0_._83_ crystals with a high selenium concentration, an orange band is clearly emphasized. At high temperatures, the probability of S evaporation from S and Se solutions is substantially increased. In [69], it was demonstrated that Se can substitute oxygen atoms in *β*–Ga_2_O_3_ with the formation of diluted Ga_2_(O_1−x_Se_x_)_3_ solutions with x ≤ 0.16, whose indirect band gap is greatly influenced by Se concentration. Thus, one can admit that the structure of both violet and green–orange PL bands in the gallium oxide layer formed by high-temperature heat treatment in air at single crystal lamellae of GaS_x_Se_1−x_ solid solutions (0.05 ≤ x ≤ 0.95) is determined by the presence of selenium impurities in *β*–Ga_2_O_3_.

Single crystals of GaS, GaSe, and their solid solutions are formed by elementary S/Se-Ga-Ga-S/Se stratified packings with interlayer spacings of ~0.3 nm [70], through which oxygen atoms may easily diffuse. Thus, at high temperatures, the formation process of *β*–Ga_2_O_3_ takes place in the entire volume of the plate. By 6 h of heat treatment (in AVH_2_O, at 900 °C) of the 35 µm thick GaS plate, a homogeneous layer of nanostructured *β*–Ga_2_O_3_ was obtained. This sample served as the photogenerating element of electron-hole pairs in a UV-C photodetector.

**Figure 9:**
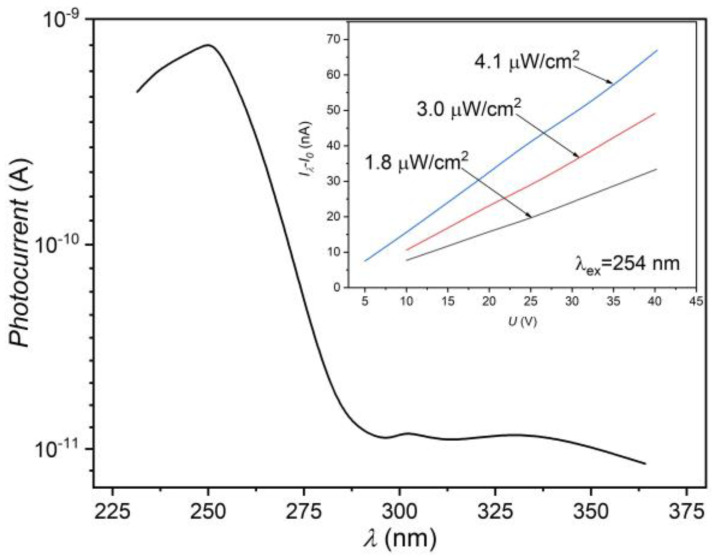
Spectral dependence of the photocurrent in a photoresistor based on the nanostructured *β*–Ga_2_O_3_ layer. Inset: current-voltage characteristic of the photoresistor.

The Au and Au/Ti thin films are used as electrode material in *β*–Ga_2_O_3_-based photodetectors [71,72,73,74]. In Ref. [75], the current-voltage characteristics are analyzed in photodetectors based on thin films of *β*–Ga_2_O_3_ with Au and Al electrodes, from which the linear dependence in the range of 0 ± 10 V under dark and illumination conditions is clearly seen for the photodetector with Al electrodes. The linearity of the current-voltage characteristic in photodetectors with Au electrodes after illumination is observed at voltages greater than ±2.14 V. The photoresistor studied in this work was obtained by vacuum deposition (10^−6^ Torr) of two Al ribbons at a distance of ~2 mm from each other. The structure thus obtained was subjected to a heat treatment at ~400 °C for 2 h in vacuum. Figure 9 shows the spectral distribution of the photocurrent upon illumination with radiation provided by a filtered Xe lamp.

As can be seen from the last figure, in the spectral range between 280 and 245 nm, the photocurrent through the photoresistor increases by more than two orders of magnitude, from ~10^−11^ to ~10^−9^ A. The maximum photocurrent is obtained for λ = 250 nm. The photoresponse of the detector at 250 nm wavelength is 0.9 nA. Figure 9 (Inset) shows the current-voltage dependencies upon excitation with a 254 nm wavelength radiation, the power density of which was 1.8, 3.0, and 4.1 µW/cm^2^. In the voltage range from 5 to 40 V, the photocurrent was found to increase linearly with the applied voltage.

## 4. Conclusions

The lamellar semiconductors in the series of GaS_x_Se_1−x_ solid solutions exhibit a bandgap in the range of 1.98–2.58 eV. By using a heat treatment of respective single crystals in AVH_2_O at temperatures between 750 and 900 °C, homogeneous layers consisting of *β*–Ga_2_O_3_ nanowires/nanosheets with micrometric lengths are obtained. Depending on the heat treatment temperature, *β*–Ga_2_O_3_ layers with monoclinic crystal lattice are obtained, but also nanocomposite layers formed by *β*–Ga_2_O_3_ and Ga_2_Se_3_/Ga_2_S_3_ crystallites with submicrometric dimensions. The direct band gap of the *β*–Ga_2_O_3_ layer depends on the composition (x) of GaS_x_Se_1−x_ solid solution and was found in the range of 4.52–4.82 eV. The *β*–Ga_2_O_3_ and *β*–Ga_2_O_3_/Ga_2_Se_3_/Ga_2_S_3_ nanocomposite layers are photoluminescent materials in the UV–blue region. Resistive detectors based on nanostructured *β*–Ga_2_O_3_ layers show high sensitivity in the UV-C region (230–270) nm. The photocurrent obtained by irradiating the surface with a 254 nm beam is higher than the dark current by more than two orders of magnitude.

## Figures and Tables

**Table 1:** Elemental composition of *β*–Ga_2_O_3_ layer on GaS_x_Se_1−x_ solid solution substrate.

Composition (x) of the Primary Compound GaS_x_Se_1−x_	Heat Treatment Temperature, °C	Heat Treatment Duration, h	Concentration, at. %		*C_Ga_*/*C_O_*
			*C_O_*	*C_Ga_*	
1.00	850	6.0	59.33	40.67	2/2.92
1.00	900	0.5	56.08	43.92	2/2.60
0.80	900	6.0	60.78	39.22	2/3.10
0.50	900	6.0	61.01	38.99	2/3.13
0.17	900	6.0	53.15	46.85	2/2.30
0.05	900	6.0	57.40	42.60	2/2.70

**Table 2:** Raman vibration modes of *β*–Ga_2_O_3_ layers on GaS_x_Se_1−x_ solid solution substrate.

No	Sulfur Concentration x in GaS_x_Se_1−x_ Solid Solution Substrate												
	x = 0.0, *t* = 750 °C		x = 0.0, *t* = 900 °C		x = 0.17, *t* = 900 °C		x = 0.5, *t* = 900 °C		x = 0.8, *t* = 900 °C		x = 1.0, *t* = 900 °C		Symm. [5]
	$\tilde{\nu }$, cm^−1^	*Int*., a.u.	$\tilde{\nu }$, cm^−1^	*Int*., a.u.	$\tilde{\nu }$, cm^−1^	*Int*., a.u.	$\tilde{\nu }$, cm^−1^	*Int*., a.u.	$\tilde{\nu }$, cm^−1^	*Int*., a.u.	$\tilde{\nu }$, cm^−1^	*Int*., a.u.	
1	73.0	10	120.0	15	114.0	22	117.0	20	115.0	31	114.0	22	*B_g_*
2	86.0	78	158.0	42	146.0	31	145.0	36	145.0	28	144.0	31	*B_g_*
3	113.5	95	179.0	67	173.0	40	172.0	179	174.0	42	171.0	40	*A_g_*
4	147.0	15	207.0	227	201.0	100	119.0	860	199.0	85	199.0	100	*A_g_*
5	186.5	93	328.0	43	322.0	33	321.0	44	324.0	27	323.0	33	*A_g_*
6	231.6	63	355.0	89	349.0	49	284.0	375	348.0	52	348.0	49	*A_g_*
7	290.0	23	422.0	85	416.0	50	414.0	428	417.0	58	415.0	50	*A_g_*
8	357.0	100	484.0	42	478.0	35	476.0	72	478.0	36	475.0	35	*A_g_*
9	384.6	19	636.0	41	630.0	36	629.0	347	628.0	30	633.0	36	*A_g_*
10			662.0	79	656.0	47	655.0	347	657.0	48	655.0	47	*A_g_*
11			773.0	109	767.0	58	762.0	467	765.0	56	765.0	58	*A_g_*

$\tilde{\nu }$—wavenumber; *t*—temperature.

## Data Availability

Not applicable.
